# Dandelion‐Like Tailorable Nanoparticles for Tumor Microenvironment Modulation

**DOI:** 10.1002/advs.201901430

**Published:** 2019-09-12

**Authors:** Qin Guo, Xi He, Chao Li, Yongqing He, Yiying Peng, Yu Zhang, Yifei Lu, Xinli Chen, Yujie Zhang, Qinjun Chen, Tao Sun, Chen Jiang

**Affiliations:** ^1^ Key Laboratory of Smart Drug Delivery Ministry of Education State Key Laboratory of Medical Neurobiology Department of Pharmaceutics School of Pharmacy Fudan University Shanghai 201203 China

**Keywords:** penetration, pH‐triggered release, tumor‐associated macrophages (TAMs) polarization, tumor therapy

## Abstract

Tumor‐associated macrophages (TAMs) constitute over 50% of the number of cells within the tumor, playing a major role in tumor progression and invasion. Remodeling the tumor immune microenvironment by modulating TAM polarization has been emerging as a new and promising therapeutic strategy. However, the high interstitial fluid pressure and dense extracellular matrix lead to insufficient penetration of nanosized therapies. To overcome this dilemma, an acid‐triggered size‐changeable nanoparticle (aptamer/acid sensitive linker crosslinked DGL/zoledronic acid, i.e., Apt@(DGL‐ZA)*_n_* NPs) with effective tumor distribution, extravasation, and penetration is designed. Dendrigraft poly‐*L*‐lysines (DGLs) which can induce tumor autophagy as mimics of natural abnormal proteins are crosslinked via a mild‐acid‐responsive linker (1,6‐bis(4‐formylbenzoyloxy) hexane). Long circulation property and tumor penetration are achieved simultaneously by catching DGLs in neutral pH while releasing them in the tumor's pH, like dandelion seeds in midair. The macrophage conditioning agent zoledronic acid (ZA) is loaded on DGLs by the charge attraction. A Tenascin‐C targeting aptamer (GBI‐10) is modified onto (DGL‐ZA)*_n_* NPs for a tumor‐homing effect. Apt@(DGL‐ZA)*_n_* NPs show both enhanced penetration in in vitro 3D triple negative breast cancer spheroids and in vivo tumor tissues. Effective macrophage regulation, enhanced tumor autophagy, and excellent in vivo antitumor efficacy are achieved, suggesting this tactic as a significant antitumor strategy.

## Graphical abstract

Remodeling the tumor immune microenvironment via modulation of tumor‐associated macrophage polarization is an effective strategy for tumor therapy, but limited by the high interstitial fluid pressure and dense extracellular matrix. A novel type of pH triggered Apt@(DGL‐ZA)*_n_* nanoparticles is developed for enhanced tumor penetration and deep macrophage polarization in vivo.

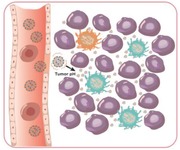

Cancer arises in the context of an in vivo tumor microenvironment (TME). TME is critical to both the initiation and maintenance of tumorigenesis.^1^ Tumor and host cells coevolve dynamically eliciting multiscale effects on many biological programs, including cellular proliferation, growth, and metabolism, as well as angiogenesis and hypoxia and innate and adaptive immunity through indirect and direct cellular interactions.^2^ Accumulating evidence strongly indicates that clinical responses to chemotherapy can be enhanced if the TME was improved simultaneously.^3^

In most solid tumors, TME is comprised of nonmalignant cells, such as cancer associated fibroblasts, endothelial cells and pericytes composing tumor vasculature, immune and inflammatory cells, bone marrow derived cells, and the extracellular matrix (ECM), establishing a complex network.^4^ Tumor‐associated macrophages (TAMs) is an important node and hub in this network, since they can be present in large quantities in cancer. TAMs may constitute over 50% of the number of cells within the tumor, as a major player involved in tumor progression.^5^ Furthermore, TAMs were regarded as a double‐edged sword, either inhibiting or promoting the tumor progression,^6^ attributed to the flexible polarization to two major phenotypes: the antitumor M1 (TAM1) and the promote tumor M2 (TAM2) during tumor progression. In the TME, TAM2 were recruited by cytokines secreted by cancer cells. In return, TAM2 can produce high amounts of promote tumor cytokines to influence tumor progression. TAM2 inhibit infiltration and function of antitumor CD8+ T‐cell, induce angiogenesis, and promote tumor cell proliferation and metastasis.^7^ Therefore, remodeling the tumor immune microenvironment via modulation of the TAM polarization has been emerging as a new therapeutic tactic recently. Multiple drugs have been developed to achieve TAM selective polarization and curative effect, including regorafenib, zoledronic acid, and nucleic acid drugs, for instance, miR‐155.^8^

Compared with conventional small‐molecule drugs, nanoparticle‐based therapeutics tend to preferentially accumulate in solid tumors through the classic enhanced permeability and retention (EPR) effect.^9^ For its favorable antineoplastic effects, nanoparticles have been explored as a promising delivery vector for TAMs polarization.^6^, ^10^ Certain curative effect was obtained via albumin‐, exosome‐,^8^ liposomal‐11 based biomimetic delivery systems,^6^ and other nanocarriers.^12^ However, the EPR‐required size also greatly limits deep penetration of the nanotherapeutics into the tumor parenchyma.^13^ It has been demonstrated that nanotherapeutics, after extravasation from the tumor vessels, are mainly restricted to the adjacent regions of tumor vasculatures due to the high IFP and dense extracellular matrix, thus, greatly compromising their therapeutic effects.^14^

To address the predicament, several strategies have been reported. For instance, cell penetrating peptide modification is one of the most promising strategies for enhancing the permeability of therapeutic agents and widely applied in abnormally high dense stroma tumor like Pancreatic ductal adenocarcinoma.^15^ Meanwhile, in this strategy, the lack of selectivity for targeting cells, and undesired tumor accumulation in vivo were reported.[qv: 15a] Rationally regulating the physiochemical properties of nanoparticles such as particle size and shape remains a challenging issue.^16^

It is reported that smaller nanoparticles generally show stronger tumor permeability because of reduced diffusional hindrance, but often suffer from inferior circulating half‐life time and tumor accumulation.^17^ One way around this dilemma is to develop a size‐changeable delivery system that could maintain large initial size for prolonged blood circulation and selective extravasation, while transforming into small particles within tumor tissues for deep penetration and effective tumor distribution. For instance, Wang et. al. established an instantaneous size‐changeable superstructure for active cisplatin delivery,^18^ confirming the significantly enhanced tumor permeability via size‐changeable delivery system. However, besides focusing chemotherapeutics delivery, a TME‐triggered size‐changeable nanoplatform for deep TAMs polarization for its tremendous potential of TME modulation in tumor treatment is still in great demand.

Herein, we reported the design of a tumor pH‐sensitive dendrigraft poly‐*L*‐lysines (DGLs) nanoparticle (NPs), which was crosslinked via tumor pH response linker (1,6‐bis(4‐formylbenzoyloxy) hexane). The linker could “catch” DGLs in neutral pH, and release them in tumor pH, just like dandelion seeds in the air, to achieve long circulation and tumor penetration simultaneously. In eukaryotes, intracellular accumulation of abnormal proteins is a principal factor to induce autophagy for degradation of these macromolecules. As bioinspired by this process, mimicking hallmarks of natural abnormal proteins is a promising strategy to create desired pharmacological activity for tumor autophagy induction. DGLs, as protein mimics, similar to endogenous polypeptides in structure and size (3.0 nm insulin or 5.5 nm hemoglobin), could also induce tumor autophagy, and therefore acts as antitumor vectors and drugs at the same time.^19^, ^20^ Strong positive polarity of DGLs can be used to attract negatively charged drugs, for instance, zoledronic acid which could repolarize TAMs and inhibit mammary carcinogenesis by targeting the mevalonate pathway.^21^ DNA aptamer GBI‐10 with Tenascin‐C targeting ability was chosen as suitable camouflage for cationic material. GBI‐10 aptamer is a type of ssDNA, selected by systematic evolution of ligands by exponential enrichment (SELEX) against tenascin‐C, which is a typical ECM protein, interacts with other ECM components (e.g., fibronectin, collagen I) and cell surface receptors and is frequently up‐regulated in most solid tumor, including pancreatic cancer and breast cancer. Due to its electronegativity, it can be easily absorbed in the polymer material with positive electricity and play as a suitable camouflage for cationic material and induce nanomedicine accumulation in breast cancer via EPR effect and tenascin‐C targeting ability.^22^ According to the original design, Apt@(DGL‐ZA)*_n_* NPs could go through the detachment of GBI‐10 and exposure of crosslinked‐DGL NPs after the accumulation in tumor tissue at Tenascin‐C‐highly expressed tumor microenvironment. Our Apt@(DGL‐ZA)*_n_* NPs showed great potential for tumor autophagy induction, TAMs repolarization, furthermore, tumor microenvironment improvement. In vivo pharmacodynamics study indicates there was no significant difference between 25% Taxol plus Apt@(DGL‐ZA)*_n_* NPs and original dose of Taxol in tumor suppressive effect, but toxicity was significantly reduced.

The preparation of the Apt@(DGL‐ZA)*_n_* NPs is illustrated in **Scheme** 1. First, we explored the synthesis of tumor pH responsive linker 1,6‐bis(4‐formylbenzoyloxy) hexane via esterification. The synthetic route and ^1^H NMR of pH responsive linker were shown in Figure S1(Supporting Information). Afterward, (DGL‐ZA)*_n_* NPs were prepared simply via self‐assembly filming‐rehydration. Optimization of the weight ratio of DGLs, cross‐linker, and zoledronic acid showed the most uniform NPs have polydispersity index of 0.068 ± 0.002 and average particle size of 123 ± 1 nm (Figure S2A, Supporting Information). To avoid damage of the toxic cationic materials and off‐target accumulation of (DGL‐ZA)*_n_* NPs in vivo, we further utilized Tenascin‐C targeting aptamer GBI‐10 as negatively charged camouflage, also tumor‐homing corona. As shown in Figure S2B (Supporting Information), we formulated the (DGL‐ZA)*_n_* NPs and GBI‐10 from 10:1 to 1:5. With the addition of negatively charged GBI‐10 aptamer, the zeta potential of Apt@(DGL‐ZA)*_n_* NPs shifted from −13.4 ± 0.7 mV to −22.4 ± 2.3 mV. The particle size increased greatly from 123 ± 1 to 201 ± 7 nm. Considering of the suitable size and particle charge for drug delivery, we optimized N/P as 10:1 with a size of 123 ± 1 nm and zeta potential as −13.4 ± 0.4 mV. The drug loading ability of Apt@(DGL‐ZA)*_n_* NPs was measured by high performance liquid chromatography to be 3.1% ± 0.3%. Variation in particle size and charge during preparation process of Apt@(DGL‐ZA)*_n_* NPs was observed (**Figure** 1B,C). Acid‐triggered size change of Apt@(DGL‐ZA)*_n_* NPs has been verified by DLS and TEM (Figure 1E, G), in which smaller particles less than 10 nm were observed at pH 6.8 (mimicking the extracellular tumor acidic microenvironment). DLS and TEM result of pH‐insensitive Apt@C(DGL‐ZA)*_n_* NPs was also displayed as control (Figure 1D,F).

**Scheme 1:**
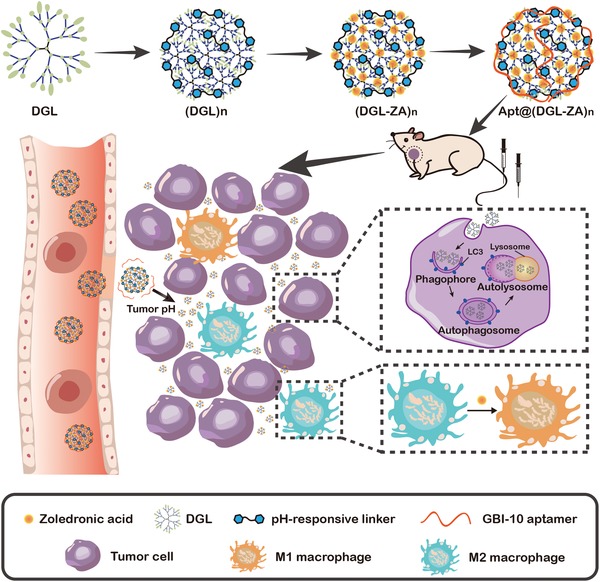
Schematic illustration of Apt@(DGL‐ZA)*_n_* NPs formulation and pH triggered tumor penetration of Apt@(DGL‐ZA)*_n_* NPs as well as effect in vivo, including macrophage polarization and autophagy of tumor cells.

**Figure 1:**
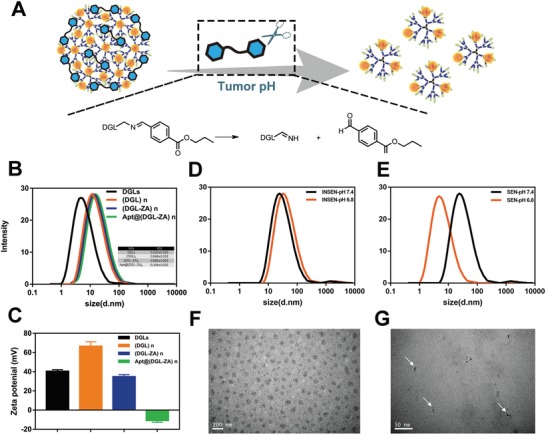
A) Schematic illustration showing the disintegration of SEN/ZOL NPs into small particles at tumor acidic pH. B) Size distribution of DGLs, (DGL)*_n_* NPs, (DGL‐ZA)*_n_* NPs, and Apt@(DGL‐ZA)*_n_* NPs; C) Zeta potential of DGLs, (DGL)*_n_* NPs, (DGL‐ZA)*_n_* NPs, and Apt@(DGL‐ZA)*_n_* NPs; D) Size distribution of Apt@C(DGL‐ZA)*_n_* NPs at pH 7.4 and pH 6.8. E) Size distribution of Apt@(DGL‐ZA)*_n_* NPs at pH 7.4 and pH 6.8; TEM images of Apt@(DGL‐ZA)*_n_* NPss at F) pH 7.4, G) pH 6.8.

Recent studies indicated that nanomedicines for cancer therapy with smaller size exhibit enhanced in vivo performance through greater tumor penetration.^18^, ^23^ As motivated by the sensitivity of Apt@(DGL‐ZA)*_n_* NPs in response to tumor extracellular pH, we investigated its potential to overcome the pathological penetration barrier. Due to the similarity in morphology and biological microenvironment to solid tumors, tumor multicellular spheroids are versatile 3D models for tumor biology.^24^ We constructed a 4T1 multicellular 3D spheroid model (MSCs) to explore whether Apt@(DGL‐ZA)*_n_* NPs have enhanced pH‐triggered tumor penetration. Apt@C(DGL‐ZA)*_n_* NPs (pH‐insensitive NPs) and cApt@(DGL‐ZA)*_n_* (scrambled‐aptamer NPs) were presented as control. Upon treatment with FITC‐labeled Apt@(DGL‐ZA)*_n_* NPs, Apt@C(DGL‐ZA)*_n_* NPs, and cApt@(DGL‐ZA)*_n_* at pH 6.8 and pH 7.4 for 4 h, MSCs were monitored under confocal laser scanning microscopy (CLSM). As shown in **Figure** 2, Z‐stack scanning showed that the green fluorescence of Apt@(DGL‐ZA)*_n_* NPs were mostly constrained in the peripheral sections of tumor spheroid at neural pH. In comparison, the penetration capability of Apt@(DGL‐ZA)*_n_* NPs at tumor pH enhanced significantly because they can rapidly disintegrate into small particles at acidic environment. Meanwhile Apt@C(DGL‐ZA)*_n_* NPs incubated at pH 6.8 for 4 h showed comparable penetration behavior to Apt@C(DGL‐ZA)*_n_* NPs at pH 7.4. This result further illustrated that the enhanced penetration capability of Apt@(DGL‐ZA)*_n_* was vested by mild‐acid‐sensitive linker. As for cApt@(DGL‐ZA)*_n_* NPs, they have similar penetrating behavior with Apt@(DGL‐ZA)*_n_* NPs at the peripheral sections of tumor spheroid, but were constrained away from the centric position of tumor spheroid. Such phenomenon might be due to the different bioeffect of random sequence aptamer (cApt) and tenascin‐C affinity aptamer GBI‐10. Due to the high affinity of GBI‐10 with tenascin‐C in the matrix of tumor spheroid, GBI‐10 could be detached from Apt@(DGL‐ZA)*_n_* NPs and expose (DGL‐ZA)*_n_* NPs to induce the deep penetration. These in vitro results suggest that our Apt@(DGL‐ZA)*_n_* NPs could perform ECM‐acid triggered penetration behavior, thus probably advantageous for in vivo tumor penetration capability.

**Figure 2:**
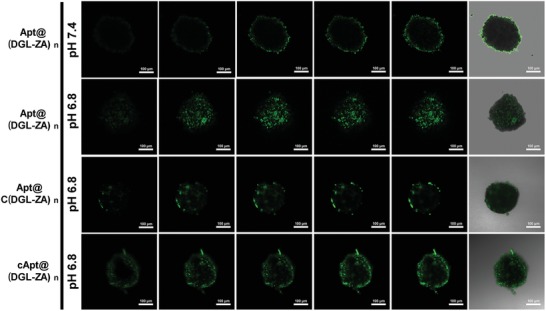
CLSM images of in vitro penetration of FITC‐labeled Ap@(DGL‐ZA)*_n_* NPs, Apt@C(DGL‐ZA)*_n_* NPs, and cApt@(DGL‐ZA)*_n_* NPs at pH 6.8 or pH 7.4 in 4T1 multicellular spheroids (MSCs). The MSCs were monitored by CLSM Z‐scanning with a 20 µm interval between consecutive images.

To characterize the tumor penetration properties of NPs in vivo, after intravenous injection with cApt@(DGL‐ZA)*_n_* NPs, Apt@C(DGL‐ZA)*_n_* NPs, and Apt@(DGL‐ZA)*_n_* NPs, respectively, tumor accumulation and penetration efficacy of BODIPY‐labeled NPs on orthotopic TNBC bearing female mice were monitored by near‐infrared imaging noninvasively. High expression of Tenascin‐C was found in tumor sections of breast cancer (Figure S3, Supporting Information). As predicted, Apt@(DGL‐ZA)*_n_* NPs and Apt@C(DGL‐ZA)*_n_* NPs showed stronger signal at TNBC site from 8 to 12 h compared with cApt@(DGL‐ZA)*_n_* NPs. 3D imaging of Apt@(DGL‐ZA)*_n_* NPs‐treated mice showed colocalization of Apt@(DGL‐ZA)*_n_* NPs to the tumor site (**Figure** 3B). After 12 h, tumors and major organs were excised for ex vivo imaging (Figure 3C). Apt@(DGL‐ZA)*_n_* NPs showed stronger signal than Apt@C(DGL‐ZA)*_n_* NPs in quantitative analysis (Figure 3D) indicates improved tumor accumulation. Such phenomenon might be due to the enhanced penetration of Apt@(DGL‐ZA)*_n_* NPs avoided congestion of nanoparticles in proximity to blood vessels. To further investigate the penetration behavior of different NPs, tumor sections were obtained from the above‐mentioned excised tumor. Then tumor vessels were labeled with anti‐CD34 antibody (green) and cell nucleus were stained with DAPI fluorescence (blue). After that, tumor sections were observed by CLSM. Consistent with Figure 3D, Apt@(DGL‐ZA)*_n_* NPs showed most accumulation in tumor tissues. Though Apt@C(DGL‐ZA)*_n_* NPs showed comparable tumor accumulation with Apt@(DGL‐ZA)*_n_* NPs, they behaved very different in the tumor region away from the blood vessels. Under large field of view (with a scale bar of 250 µm) Apt@C(DGL‐ZA)*_n_* NPs were usually found constrained in proximity to blood vessels, while Apt@(DGL‐ZA)*_n_* NPs could distal penetrate from the vessel toward the interior of the tumor because size limited infiltration into tight intercellular junctions without additional assistance (Figure 3E). These combined in vitro and in vivo results suggest potential benefits of Apt@(DGL‐ZA)*_n_* NPs in TAMs repolarization and tumor treatment.

**Figure 3:**
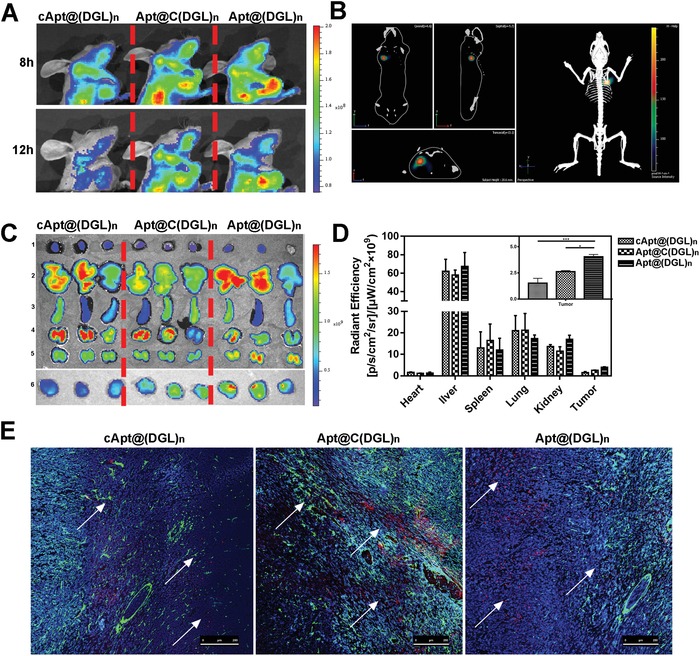
A) In vivo 2D fluorescent images of cApt@(DGL‐ZA)*_n_* NPs, Apt@C(DGL‐ZA)*_n_* NPs, and Apt@(DGL‐ZA)*_n_* NPs post 8 h or 12 h i.v. injection. B) In vivo 3D fluorescent image of Apt@(DGL‐ZA)*_n_* NPs. C) Biodistribution of cApt@(DGL‐ZA)*_n_* NPs, Apt@C(DGL‐ZA)*_n_* NPs, and Apt@(DGL‐ZA)*_n_* NPs in heart 1), liver 2), spleen 3), lung 4), kidney 5), and tumor 6) post 24 h i.v. injection. D) Quantified fluorescence of (C). Data points represent mean ± s.d. (*n* = 5). * and *** denote *p* < 0.05 and *p* < 0.001. E) Confocal images of tumor tissues post 24 h i.v. injection of cApt@(DGL‐ZA)*_n_* NPs, Apt@C(DGL‐ZA)*_n_* NPs, and Apt@(DGL‐ZA)*_n_* NPs. Blue: DAPI stained nucleus, green: CD34, red: BODIPY‐labeled NPs.

To explore the capacity of Apt@(DGL‐ZA)*_n_* NPs in tumor autophagy activation and TAMs repolarization in vitro, the following evaluation was investigated. First, we employed acridine orange (AO), an autophagy probe to investigate the intensity of different NPs‐inducing autophagy at pH 6.8 and pH 7.4 in 4T1 cell line. The maximum red autolysosome was caused by Apt@(DGL‐ZA)*_n_* NPs at pH 6.8, which was comparable with the positive control rapamycin (**Figure** 4A). Such phenomenon was clearly associated with the disintegration of Apt@(DGL‐ZA)*_n_* NPs in acid environment. After calculated by fluorescence‐activated cell sorting (FACS), the highest red/green ratio was observed in Apt@(DGL‐ZA)*_n_* NPs treated 4T1 cells, reflecting the highest autophagy‐inducing activity (Figure 4B). The molecular biology evidence on the conversion of microtubule‐associated protein light chain (LC3) from LC3‐I to LC3‐II after their location at autophagosomal membranes was confirmed by western blot. The highest expression level of LC3‐II was also observed at Apt@(DGL‐ZA)*_n_* NPs in pH 6.8 (Figure 4C). All these data showed that Apt@(DGL‐ZA)*_n_* NPs induce the strongest tumor autophagy. Afterward, we investigated the capacity of different NPs in TAMs repolarization by FACS. Fresh mouse peritoneal macrophage was harvested according to previously reported method. After activated by interleukin 4 (IL‐4) for 24 h, polarized M2 macrophages were incubated with different NPs and zoledronic acid at pH 6.8 for 12 h. Then the F4/80+ (macrophage specific antigen) cell subpopulations was chosen to evaluated the radio of M1/M2 marcrophage. In control group, radio of M1(CD16/32+)/M2(CD206+) marcrophage was 0.94. Treatment with IL‐4 decreased the ratio to 0.76 while treatment with zoledronic acid increased it to 1.33, corroborated the repolarization ability of ZA. Significantly, the ratio of M1/M2 was increased to 2.67 and 2.97 after the treatment of C(DGL‐ZA)*_n_* NPs and (DGL‐ZA)*_n_* NPs, respectively. There was no significant difference between C(DGL‐ZA)*_n_* NPs and (DGL‐ZA)*_n_* NPs in at the cellular level probably because nanoparticle tissue penetration ability has nearly no effect on its cellular uptake (Figure 4D). The molecular biology evidence on the repolarization of M2 macrophages was confirmed by western blot (Figure 4E). Therefore, it is necessary to explore the macrophages repolarization capacity NPs in vivo. Influence of aptamer was not considered for the inadequate expression of matrix proteins at the cellular level.

**Figure 4:**
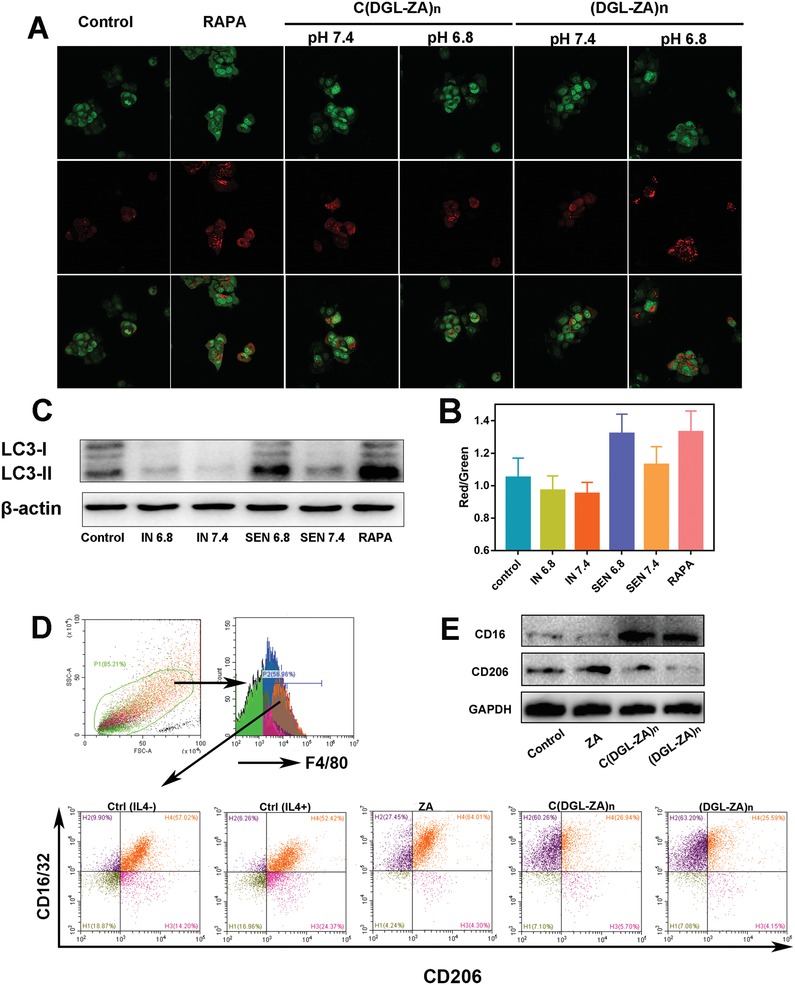
A) CLSM images and B) FACS analysis of AO‐stained 4T1 cells after incubation with Apt@(DGL‐ZA)*_n_* NPs, Apt@C(DGL‐ZA)*_n_* NPs and rapamycin as positive control at pH 6.8 and pH 7.4 for 24 h, respectively. C) Western blot assay of autophagy related LC3 protein expression in 4T1 cells after treatment with Apt@(DGL‐ZA)*_n_* NPs, Apt@C(DGL‐ZA)*_n_* NPs and rapamycin as positive control at pH 6.8 and pH 7.4 for 24 h, respectively. D) Gating strategy to determine frequencies of macrophages of tumor tissues and the ratio of M1(CD16/32+)/M2(CD206+) macrophages in total macrophages (F4/80+) after treatmen of PBS, IL4, and different NPs(preincubated with IL4). E) Western blot assay of repolarization of M2 macrophage.

Since in vitro cytotoxicity of NPs was investigated by methyl thiazolyl tetrazolium assay (Figure S4, Supporting Information) and a lower IC_50_ was observed on macrophages (0.213 ± 0.0525 µg mL^−1^) than tumor cells (4.598 ± 1.409 µg mL^−1^), we constructed orthotopic TNBC bearing female mice model using 4T1 cells to evaluate the antitumor efficacy of different NPs in vivo. Frequency and order of administration of paclitaxel (PTX) and NPs were shown in **Figure** 5A. Tumor volumes were measured every 2 d (Figure 5B). Mice body weights were recorded every 2 d to evaluate the general toxicity (Figure 5C). Initially, alternating NPs and half‐dosage of Taxol (5 mg kg^−1^) were applied, a remarkable smaller tumor volume was observed on Apt@(DGL‐ZA)*_n_* NPs, compared with other NPs and regular dose of Taxol (10 mg kg^−1^). Therefore, we further reduced the dosage of Taxol (2.5 mg kg^−1^) to explore if the effect of reducing toxicity and increasing efficiency of Apt@(DGL‐ZA)*_n_* NPs is competent. Surprisingly, the antitumor efficacy of this group was equivalent to the original dose of Taxol. Tumor growth‐curve of representative groups (red frame) were shown separately (Figure 5D). All the NPs‐treated groups showed less weight loss. No significant systemic toxicity was observed according to hematoxylin & eosin staining results (Figure S5, Supporting Information).

**Figure 5:**
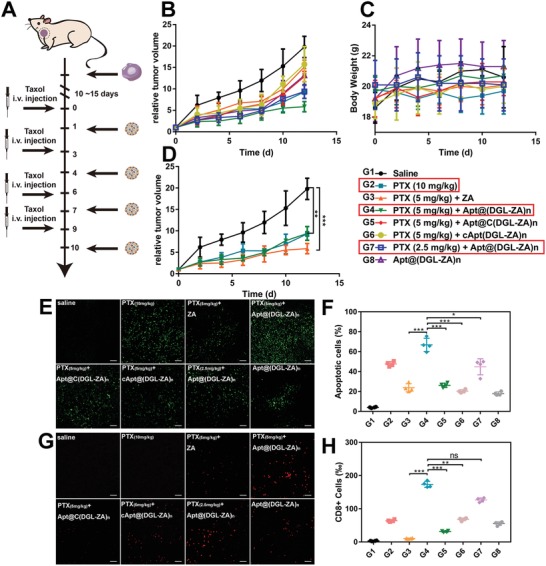
Antitumor efficacy of G1‐G8 (PTX (10 mg mL^−1^), PTX (5 mg mL^−1^) +ZA, PTX (5 mg mL^−1^) +Apt@(DGL‐ZA)*_n_* NPs, PTX (5 mg mL^−1^) +Apt@C(DGL‐ZA)*_n_* NPs, PTX (5 mg mL^−1^) +cApt@(DGL‐ZA)*_n_* NPs, PTX (2.5 mg mL^−1^) + Apt@(DGL‐ZA)*_n_* NPs, and Apt@(DGL‐ZA)*_n_* NPs) on TNBC female mice. A) Dosage regimen of PTX and NPs. B) Tumor volume changes and C) body weight after i.v. administration of different NPs and different concentration Taxol. D) Tumor growth curve of representative groups (red frame). E) histological images of 4T1 tumor xenografts of treated groups using the TUNEL assay. Green: apoptosis cells. F) Quantification of (C). G) Representative immunofluorescence images of treated groups. Red:CD8+ T cells. H) Quantification of (G). Data points represent mean ± s.d. (*n* = 4).

After the fourth dose course of therapy, the mice were sacrificed to obtain tumor tissues. Terminal deoxynucleotidyl transferase‐mediated dUTP nick‐end labeling (TUNEL) assay was carried out to detect apoptosis (Figure 5E). The green signals (FITC labeled dUTP) stained area indicated apoptosis site in tumor as they positioned the extensive DNA degradation. Samples from Apt@(DGL‐ZA)*_n_* NPs + 5 mg kg^−1^ PTX showed the most extensive apoptotic cells .

Recently, the mechanistic interplay between the TAMs and T cells has attracted attention and the modulation of TAMs can be potential avenue for boosting the efficacy of immunotherapy. For instance, TAM2 secretes transforming growth factor‐β and induces regulatory T cells (Treg), and the increased proportion of Treg leads to the inhibition of the CD8+ T‐cell responses. Conversely, if we successfully repolarized M1 macrophages into M2 macrophages, CD8+ T‐cells would be recruited to tumor tissue.^25^ Therefore, tumor tissues after treatment were stained with CD8 red signals. Samples from Apt@(DGL‐ZA)*_n_* NPs + 5 mg kg^−1^ PTX and Apt@(DGL‐ZA)*_n_* NPs + 2.5 mg kg^−1^ PTX showed the most extensive CD8+ T‐cells indicated the most effective immune activation in tumor (Figure 5G). The results of immunofluorescence were quantitative analyzed (Figure 5H).^26^

After antitumor efficacy of different NPs in vivo were investigated. We obtained tumor sections from pharmacodynamics experiment to investigate the capacity of different NPs in tumor autophagy activation and TAMs repolarization in vivo. Immunofluorescence staining of LC3 (purple signal) were carried out to confirm the activation of the autophagy effect. The strongest autophagy effect was observed in PTX (5 mg mL^−1^) +Apt@(DGL‐ZA)*_n_* NPs (**Figure** 6A), semiquantitative results were calculated by imageJ (Figure 6B).

**Figure 6:**
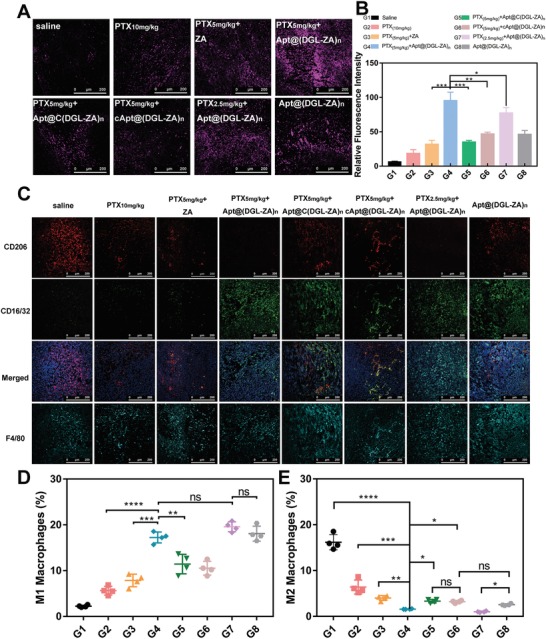
Autophagy activation and TAMs repolarization of B) G1‐G8 on 4T1 TNBC female mice. Representative immunofluorescence images of treated groups. A) Purple:LC3; C) Red: CD206; Green: CD16/32; Cyan: F4/80; (D) and (E) are quantification of (C). Data points represent mean ± s.d. (*n* = 4).

Afterward, we demonstrated the in vivo immunomodulatory effects of NPs by staining universal biomarkers of different phenotypic macrophage. CD16/32(green signal) represents M1 macrophages, CD206(red signal) represents M2 macrophages, and F4/80(cyan signal) represents all the macrophage. As has been reported, paclitaxel also could activate M1 macrophages, but the interaction with Apt@(DGL‐ZA)*_n_* NPs significantly enhanced this effect. Apt@C(DGL‐ZA)*_n_* NPs and cApt@(DGL‐ZA)*_n_* NPs showed uneven and incomplete TAMs polarization, which is consistent with their weak retention or penetration in tumor region.

In summary, we developed a pH triggered size changeable drug delivery system with effective tumor distribution, extravasation, and penetration profile. The novel Apt@(DGL‐ZA)*_n_* NPs selectively accumulated in tumor site via favorable size in systemic circulation and performed increased tumor penetration ability by the disintegration after exposed to the acidic tumor microenvironment. Enhanced drug distribution in tumor site was observed both in vitro and in vivo. With the loading of zoledronic acid and a combined application of taxol, favorable tumor microenvironment modulation efficacy was both observed in vitro and in vivo. The toxicity of Taxol in vivo was decreased. In the light of these results, the unique pH‐trigger size changeable nanoparticle with tumor penetration could serve well as a promising strategy for tumor treatment.

## Conflict of Interest

The authors declare no conflict of interest.

## Supporting information

Supplementary
