# Supplementary material for: Dandelion‐Like Tailorable Nanoparticles for Tumor Microenvironment Modulation
Source: Adv Sci (Weinh). 2019 Sep 12;6(21):1901430. doi: 10.1002/advs.201901430 (PMC6839635; doi:10.1002/advs.201901430)
Supplement: Supplementary file 1 — Supplementary [file ADVS-6-1901430-s001.pdf]

Copyright WILEY-VCH Verlag GmbH & Co. KGaA, 69469 Weinheim, Germany, 2019.

Supporting Information

for *Adv. Sci.*, DOI: 10.1002/advs.201901430

Dandelion-Like Tailorable Nanoparticles for Tumor Microenvironment Modulation

*Qin Guo, Xi He, Chao Li, Yongqing He, Yiying Peng, Yu Zhang, Yifei Lu, Xinli Chen, Yujie Zhang, Qinjun Chen, Tao Sun, and Chen Jiang**

Supporting Information

Dandelion-like Tailorable Nanoparticles for Tumor Microenvironment Modulation

*Qin Guo‡, Xi He‡, Chao Li, Yongqing He, Yiying Peng, Qinjun Chen, Yu Zhang, Yifei Lu, Xinli Chen, Yujie Zhang , Tao Sun and Chen Jiang **

Contents:

Figure S1: Synthetic route and ^{1}H NMR of pH responsive linker (1,6-bis(4-formylbenzoyloxy) hexane);

Figure S2: Formulation with different molar ratio of DGL, crosslinker and zoledronic acid via filming-rehydration method; size distribution and zeta potential of different N/P ratio of aptamer modification;

Figure S3: tenascin-C expression in 4T1 breast tumor tissues;

Figure S4: cytotoxicity of Apt@(DGL-ZA)$_n$ NPs against RAW 264.7 cells or 4T1 cells; cytotoxicity of Apt@(DGL)$_n$ NPs, Apt@(DGL-ZA)$_n$ NPs, Apt@C(DGL-ZA)$_n$ NPs, and cApt@(DGL-ZA)$_n$ NPs against HEK 293 cells;

Figure S5: H&E staining of saline, PTX(10 mg/kg), PTX(5 mg/kg) + ZOL, PTX(5 mg/kg) + Apt@(DGL-ZA)$_n$ NPs, PTX(5 mg/kg) + Apt@C(DGL-ZA)$_n$ NPs, PTX(5 mg/kg) + cApt@(DGL-ZA)$_n$ NPs, PTX(2.5 mg/kg) + Apt@(DGL-ZA)$_n$ NPs and Apt@(DGL-ZA)$_n$ NPs after treatment .

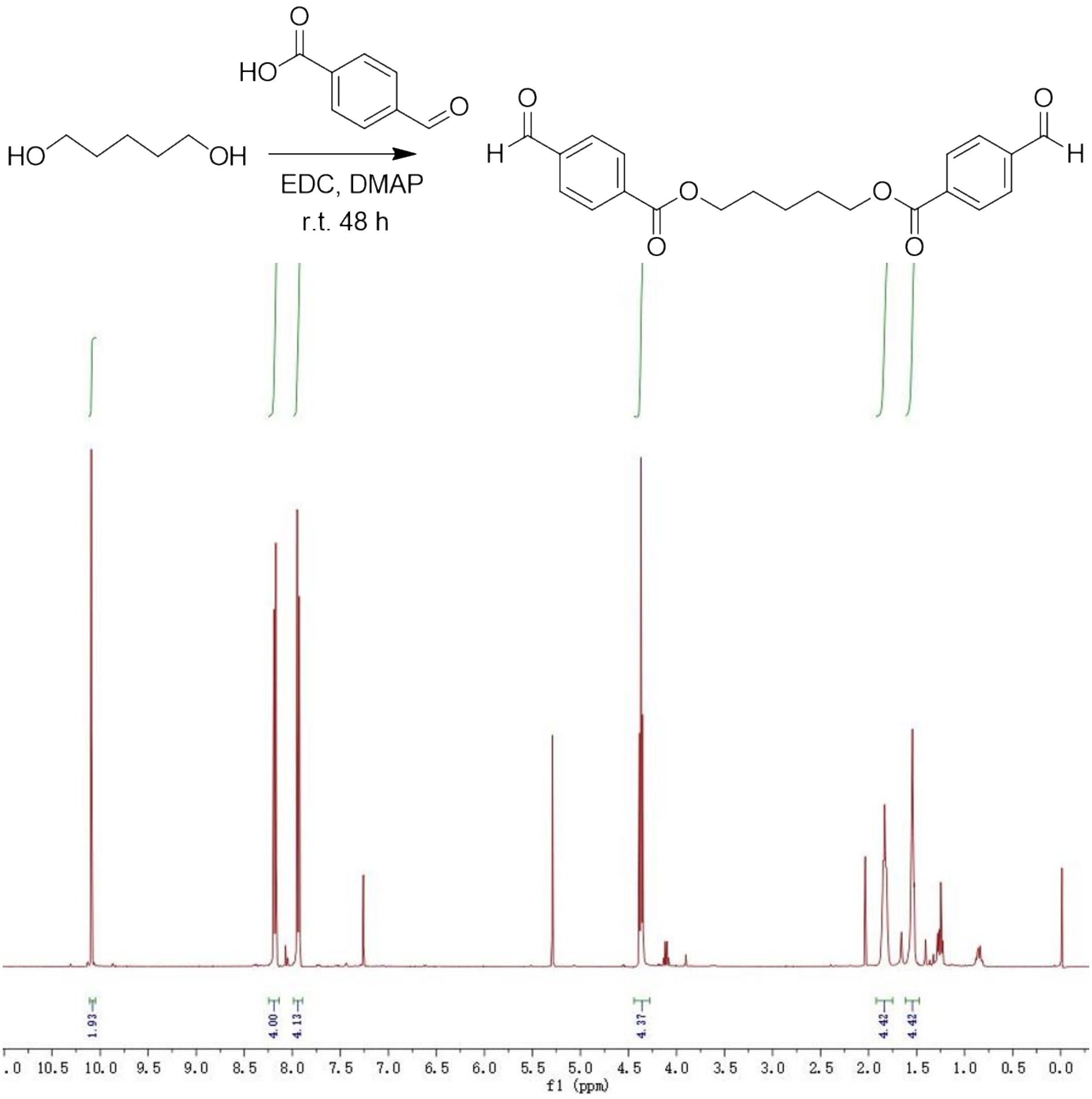

Figure S1. The synthetic route and ^{1}H NMR of pH responsive linker (1,6-bis(4-formylbenzoyloxy) hexane).

A

Entry	DGL:crosslinker: ZOL	Size (nm)	PDI
1	10:1:1	15±1	0.266±0.012
2	5:1:1	23±2	0.200±0.008
3	2:1:1	44±5	0.340±0.017
4	1:1:1	51±10	0.412±0.032
5	1:2:1	64±7	0.307±0.025
6	1:5:1	87±2	0.068±0.002
7	1:10:1	178±5	0.158±0.005
8	1:20:1	410±10	0.204±0.009

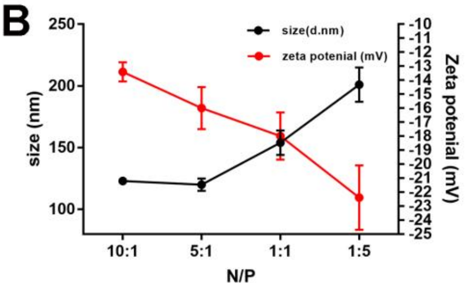

Figure S2. A) Formulation with different molar ratio of DGL, crosslinker and zoledronic acid via filming-rehydration method. B) Size distribution and zeta potential of different N/P ratio of aptamer modifcation

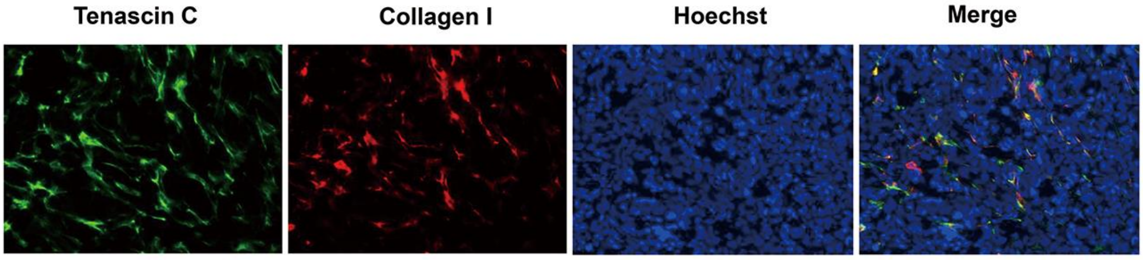

Figure S3. Tenascin C expression in 4T1 breast tumor tissues.

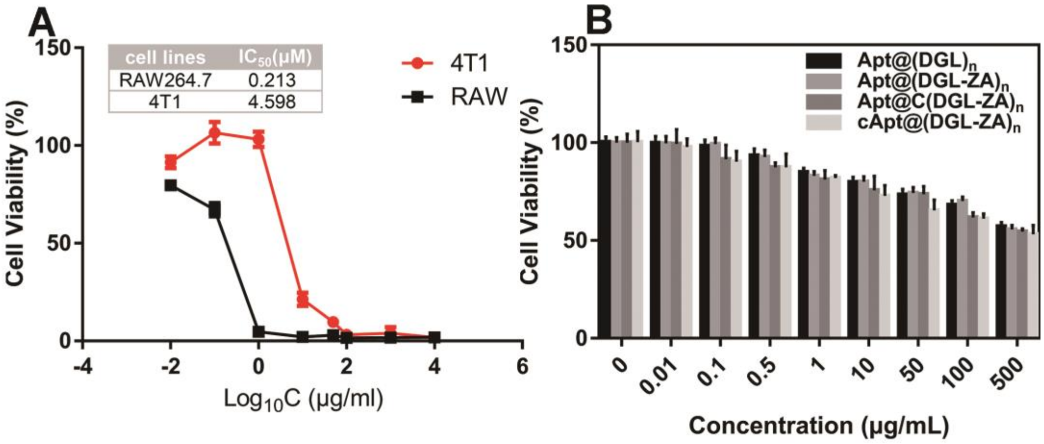

Figure S4. A) Cytotoxicity of Apt@(DGL-ZA)$_n$ NPs against RAW 264.7 cells or 4T1 cells. B) Cytotoxicity of Apt@(DGL)$_n$ NPs, Apt@(DGL-ZA)$_n$ NPs, Apt@C(DGL-ZA)$_n$ NPs, and c Apt@(DGL-ZA)$_n$ NPs on HEK 293 cells.

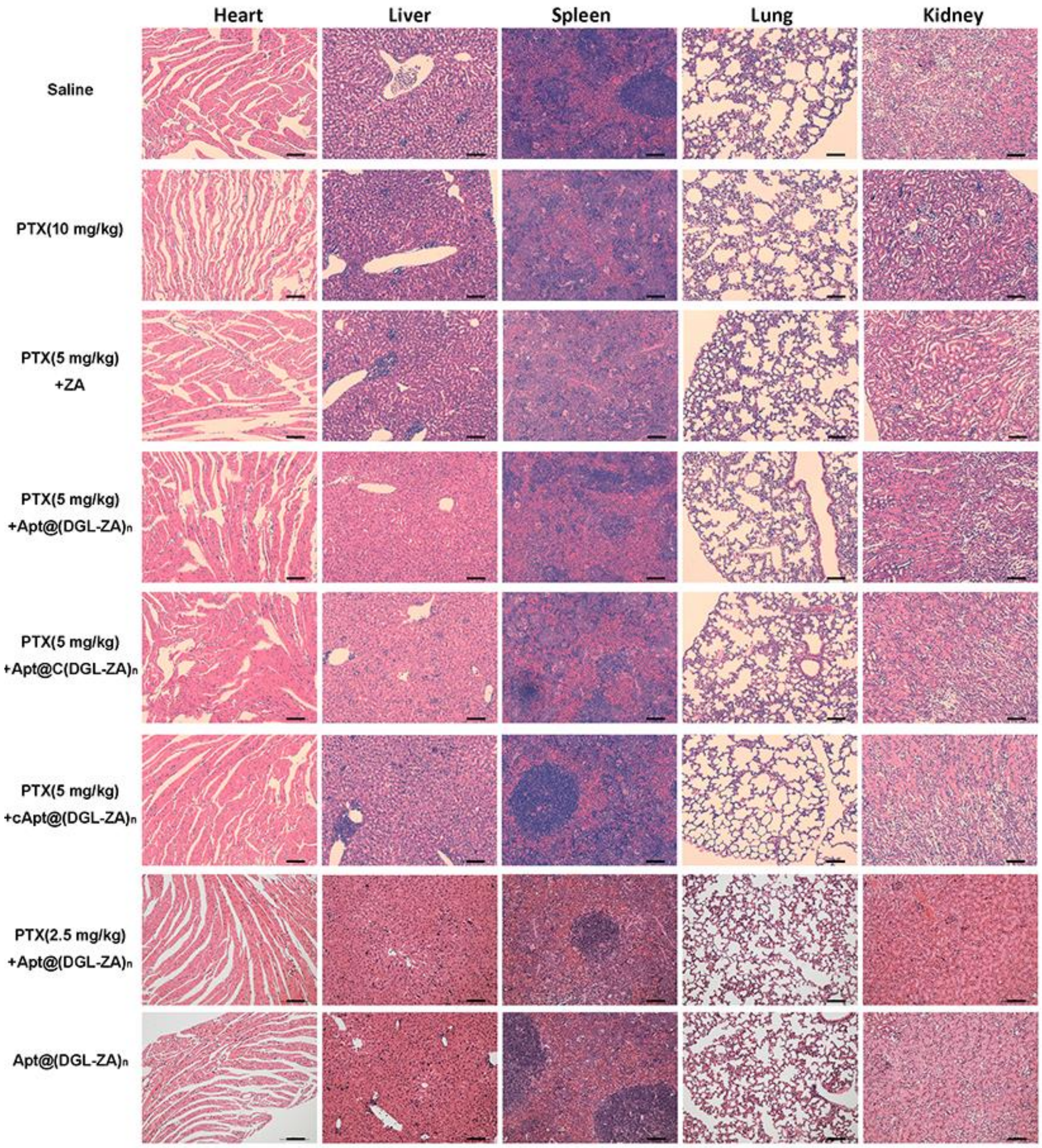

Figure S5. H&E staining of saline, PTX (10mg/mL), PTX (5mg/mL) +ZA, PTX (5mg/mL) +Apt@(DGL-ZA)$_n$ NPs, PTX (5mg/mL) +Apt@C(DGL-ZA)$_n$ NPs, PTX (5mg/mL) +cApt@(DGL-ZA)$_n$ NPs, PTX (2.5mg/mL) + Apt@(DGL-ZA)$_n$ NPs and Apt@(DGL-ZA)$_n$ NPs after treatment.

Detailed Experimental Section

Chemicals and Materials: 1,5-Pentanediol, 4-formylbenzoic acid, 4-dimethylaminopyridine and 1-(3-dimethylaminopropyl)-3-ethylcarbodiimide hydrochloride, were ordered from Energy Chemical (Shanghai, China). Dendrigraft poly-*L*-lysine (DGL, generation = 3, bearing with 123 primary amino groups, 6 nm diameter, Mw. 22000) was from COLCOM (Clapiers, France). Zoledronic acid (MB1329) was purchased from Dalian Meilun Biotechnology Co., Ltd. (Dalian, China). All organic solvents were purchased from Sinopharm Chemical Reagent Co., Ltd (Shanghai, China). BODIPY® FL NHS Ester (succinimidyl ester) and was obtained from Life Technologies Corporation (Carlsbad, USA). All antibodies were purchased from Abcam (Cambridge, England). Roswell Park Memorial Institute (RPMI)-1640 medium and fetal bovine serum (FBS) were from Gibco-Thermo Fisher Scientific (Massachusetts, USA). OCT embedding medium was purchased from Sakura Tissue-Tek® (Torrance, CA, USA). Cell lysis buffer for Western and IP were commercially available from Beyotime Biotechnology (Shanghai, China). TUNEL assay kit was bought from KeyGEN BioTECH (Nanjing, China).

Cell Lines: HEK 293 cells (derived from human embryonic kidney) and 4T1 cells were kindly provided by Stem Cell Bank, Chinese Academy of Sciences. All the cells were cultured in RPMI-1640 medium containing 10% FBS, at 37 ℃ under 5% CO_2 humidified atmosphere.

Animals: Famale bal b-c mice (20-25 g) were provided by the Experimental Animals Department of Fudan University. All animal experiments were conducted in accordance with guidelines evaluated and approved by Fudan University Institutional Animal Care and Use Committee (IACUC). The 4T1 TNBC xenograft tumor model was established by orthotopic injection of 1×10^6 4T1 cells in 100 μL serum-free RPMI-1640 medium into the mammary fat pad of mice.

Synthesis of pH-Responsive Linker (1,6-bis(4-formylbenzoyloxy) hexane): 1,5-Pentanediol (1.042 g,10 mmol) and 4-formylbenzoic acid (3.002 g, 20 mmol) were firstly dissolved in

ethyl acetate, 4-dimethylaminopyridine (DMAP, 0.122 g,1 mmol) and 1-(3-dimethylaminopropyl)-3-ethylcarbodiimide hydrochloride (EDC, 1.92 g, 10 mmol) were added and stirred for 48 h at room temperature. The crude product was purified by column chromatography using dichloromethane/methanol (10:1, v:v) to obtain a white solid (3.516 g, 87%) as the product. Disuccinimidyl suberate was applied as pH insensitive control.

Preparation of Apt@(DGL-ZA)$_n$ NPs: DGL was dissolved in methyl alcohol, and pH responsive linker was dissolved in DCM. The above two solutions were mixed evenly, followed by the removal of the solvents by rotary vacuum evaporator in a single-mouth flask with round bottom. Zoledronic acid aqueous solution was added into the flask. The flask was then putted in ultrasound for half an hour to obtain SEN/ZOL NPs. Aptamer solution at a certain N/P molar ratio (10:1 to 1:5) was added into SEN/ZOL NPs, vortexed for 30 s and maintained for another 30 min to obtain Apt@(DGL-ZA)$_n$ NPs.

BODIPY-labeled DGL was synthesized by click reaction between the amino on DGL and BODIPY® FL NHS ester (succinimidyl ester). All the BODIPY labeled NPs were prepared with BODIPY-labeled DGL.

Size, Zeta Potential and Morphology of Different NPs: Different NPs and DGL were analyzed by Malvern laser particle size analyzer to measure zeta potential and particle size. The morphologies of Apt@(DGL-ZA)$_n$ NPs in pH 7.4 and pH 6.8 were visualized using TEM (TecnaiTM G2 spirit BioTWIN, Hillsboro, USA).

Drug Loading Rate of Different NPs: The drug loading rate of different NPs were measured by HPLC at 215 nm with 10% ACN and 90% aqueous solutions (containing 0.5% tetraethylammonium hydrogensulphate, 1.6% Na_2HPO_4, 12 μM EDTA-2Na and 0.11 mM NaOH).

Preparation of 4T1 Spheroids: 4T1 TNBC tumor spheroids were prepared by natural concave deposition technique. Briefly, 2% Agarose was dissolved in RPMI-1640 medium by microwave, then maintain at 80 ℃ for 30 min for swelling. After that, the medium was sterilized by autoclaving at 121 ℃ for 30 min. 150 μL/well medium was then added into 48-well plate while still hot, then left in clean bench until condensation complete (about 30-40 min). Afterwards, 400 μL RPMI-1640 medium containing 10% FBS and 4000 cells were pipetted onto the concave surface formed by natural condensation and incubated at 37 °C with 5% CO_2 for at least one week (avoid changing medium during the process), until visible to the naked eyes. The tumor spheroids were then transferred to 24-well plate using 1 mL plastic-tips for treatment.

Confocal Microscopy of 4T1 TNBC Tumor Spheroids: The 4T1 TNBC tumor spheroids were incubated with BODIPY-labeled Apt@C(DGL-ZA)$_n$ NPs and Apt@(DGL-ZA)$_n$ NPs at equal concentration of 500 μg/mL BODIPY for 4 h. After incubation, spheroids were washed with ice-cold Hank's for three times and fixed with 4% formaldehyde for 30 min. The NPs treated spheroids were then subjected to confocal fluorescence microscope (IX2-RFACA, Olympus, Osaka, Japan) for 3D analysis.

In Vivo Biodistribution Study and Tumor Penetration Evaluation: In vivo imaging study of NPs distribution was performed on 4T1 orthotopic TNBC model. Mice were i.v. injected with BODIPY-labeled cApt@(DGL-ZA)$_n$ NPs, Apt@C(DGL-ZA)$_n$ NPs and Apt@(DGL-ZA)$_n$ NPs at equivalent BODIPY does of 0.6 mg/kg. The mice were anesthetized and visualized (IVIS Spectrum imaging system, Caliper Perkin Elmer, Waltham, USA) at Ex/Em 630/650 nm at 4, 8, and 12 h after the injection.

At 12 h, the TNBC tumor-bearing mice were anesthetized and perfused with 4% paraformaldehyde and major organs were excised for *ex vivo* imaging, then tumor tissues were sequentially immersed in 4% paraformaldehyde for 24 h, 10% sucrose solution for 12 h, 20% sucrose solution for 24 h and 30% sucrose solution for 24 h. The tumor tissues were then

frozen in OCT embedding medium at −80 °C and sectioned at 20 μm thickness (Leica, CM1900, Wetzlar, Germany). The frozen sections were immunofluorescence stained with anti-CD34 and DAPI for confocal microscopy analysis (Leica TCS SP8 STED, Wetzlar, Germany).

Acridine Orange (AO) Staining: 4T1 cells were seeded at a density of 3×10^3 cells/well to glass-bottomed confocal dishes. After 24 h, the RPMI-1640 complete medium was removed, then Apt@$(DGL\text{-}ZA)_n$ NPs or Apt@C$(DGL\text{-}ZA)_n$ NPs (120 μg/mL) were added into complete medium at pH 7.4 or pH 6.8, respectively. Meanwhile, Rapamycin was added at a final concentration of 50 nM at the same time as a positive control. After incubating for 24 h, 4T1 cells were stained with 1 μM AO for 10 min at 37 °C, then washed with Hank's thrice. The cells were observed with CLSM (Carl Zeiss LSM710, Wetzlar, Germany) with emission at 530 and 640 nm (excitation at 488 nm).

Determination of Mean Red/Green Fluorescence Ratio in AO-stained Cells by Flow Cytometry: 4T1 cells were seeded in glass-bottomed confocal dishes, at a density of 3×103 cells/well. When achieving 70-80% confluence, the RPMI-1640 complete medium was removed, then Apt@$(DGL\text{-}ZA)_n$ NPs or Apt@C$(DGL\text{-}ZA)_n$ NPs (120 μg/mL) were added into complete medium at pH 7.4 or pH 6.8, respectively. Meanwhile, Rapamycin was added at a final concentration of 50 nM at the same time as a positive control. After incubating for 24 h, 4T1 cells were stained with 1 μM AO for 10 min at 37 °C, then washed with Hank's for 3 times. Subsequently, cells were removed from the plate with trypsin-EDTA, and collected in Hank's. Green (530 nm) and red (650 nm) fluorescence emission from 5×104 cells illuminated with blue (488 nm) excitation light was measured via flow cytometry. The mean red/green fluorescence ratio of cells was calculated by FlowJo V10 software. All the experiments were performed in quadruplicate to obtain standard deviation.

Determination of TAMs Repolarization by Flow Cytometry In Vitro: Fresh mouse peritoneal macrophage was harvested as previously reported method.[1] The mouse was intraperitoneal injected with 1 mL 4% thioglycolate broth 3 days before sacrificed. The body was disinfected by immersing in 75% ethanol, then intraperitoneal injected with 2 mL DMEM medium followed by full massage for 5 min. The withdrawn DMEM medium containing fresh peritoneal macrophage could be maintained in CO_2 incubator. The harvested macrophages were seeded in 6-well culture plates at a density of 1×10^4 cells/well. When achieving 70-80% confluence, macrophages were incubated with IL-4 (20 ng/mL). After 48h incubation, cells were washed with Hank's thrice, and then incubated with or without (as negative control) formulations equivalent to 50 ng/ml zoledronic acid. After 12 h incubation, cells were removed from the plate with trypsin-EDTA and collected into the 1.5 mL centrifugal tube, and then resuspended with 200 μL PBS. Every tube was blocked with 5 μL 0.5 mg/mL BSA for 10min, and subsequently incubate with 0.025 μL F4/80 antibody (FITC labeled, ab60343), 0.125 μL CD16/32 antibody (Phycoerythrin labeled, ab218781) and 2 μL CD206 antibody (APC labeled, ab195192) for 20min, then analyzed by flow cytometry.

Western Blot Assay: 4T1 cells were incubated with $(DGL\text{-}ZA)_n$ NPs or $(DGL\text{-}ZA)_n$ NPs (120 μg/mL) for 24 h. Cells were collected in centrifugal tube and gently washed with ice-cold Hank's and homogenized in Cell lysis buffer for Western and IP (1 mM PMSF was extra added) for 15 min, followed by centrifugation (12000 g, 4 °C) for 5min and supernatant transfer to a new centrifugal tube. The protein concentration of cell sample was measured by BCA Protein Assay kit. Then cell lysates containing equivalent amounts of protein (50 μg/hole) were separated by 12% SDS-PAGE electrophoresis at 100 V for 1.5 h and transferred to PVDF membranes. After being blocked with 5% (w/v) skim milk for 1 h at room temperature, the PVDF membranes were incubated overnight with LC3 antibody (1:500, N/N) in Tris-Buffered-Saline with Tween-20 (TBST) at 4 °C. After washed with TBST for 3 times ,10 min each time and incubated goat anti-rabbit or anti-mouse secondary antibody

(1:500) conjugated with HRP for 1 h at room temperature. Then second antibody solution were removed. The membranes were washed twice for 10 minutes with TBST buffer. Finally, the target proteins were visualized with ECL plus reagent according to the manufacturer's instructions and imaged in a Bio-Rad17 ChemiDoc™ XRS+ system.

In Vivo Anti-Tumor Efficacy Study: At the 10th day after implantation, TNBC bearing mice were randomly divided into eight groups (n = 8 each group) according to the size of the tumor. Saline or high (10 mg/kg), medium (5 mg/kg), low (2.5 mg/kg) dose of PTX were administered on day 0, 3, 6 and 9. Saline or Zoledronic acid, Apt@$(DGL\text{-}ZA)_n$ NPs, Apt@C$(DGL\text{-}ZA)_n$ NPs, cApt@$(DGL\text{-}ZA)_n$ NPs were administered on day 1, 4, 7 and 10. Body weights of mice were recorded every other day. Tumor volume was calculated as $a \times b^2/2$, where a is the largest and b is the smallest diameter. Mice were sacrificed on day 12 and major organs including heart, liver, spleen, lung, kidney and tumor were excised and fixed in 4% paraformaldehyde. The apoptotic cells in tumor tissues were evaluated using transferase-mediated deoxyuridine triphosphate-biotin nick end labeling (TUNEL) assay, according to commercial instructions. Histological changes in main organ tissues were evaluated using hematoxylin and eosin (HE) staining.

Immunofluorescence Analysis of LC3, CD8, CD16/32, F4/80 and CD206: At the 12th day after the start of administration, the TNBC tumor-bearing mice were anesthetized and perfused with 4% paraformaldehyde and tumor tissues were sequentially immersed in 4% paraformaldehyde for 24 h, 10% sucrose solution for 12 h, 20% sucrose solution for 24 h and 30% sucrose solution for 24 h. The tumor tissues were then frozen in OCT embedding medium at −80 °C and sectioned at 20 μm thickness (Leica, CM1900, Wetzlar, Germany). The frozen sections were immunofluorescence stained with LC3 antibody (1:1000, ab48394) or CD 8 antibody (1:1000, ab22378) at 4 ℃ for 12 h, followed by fluorescence labeled secondary antibody at room temperature for 1 h. The frozen sections were stained CD16/32

antibody, CD206 antibody or F4/80 antibody directly. All images were taken by confocal microscopy analysis (Leica, CM1900, Wetzlar, Germany).

Reference

[1] V. Baron-Bodo, P. Doceur, M. L. Lefebvre, K. Labroquère, C. Defaye, C. Cambouris, D. Prigent, M. Salcedo, A. Boyer, A. Nardin, *Immunobiology.* **2005**, 210, 267.
